# Valid and Reliable Barbell Velocity Estimation Using an Inertial Measurement Unit

**DOI:** 10.3390/ijerph18179170

**Published:** 2021-08-31

**Authors:** Steffen Held, Ludwig Rappelt, Jan-Philip Deutsch, Lars Donath

**Affiliations:** Department of Intervention Research in Exercise Training, Institute of Exercise Training and Sport Informatics, German Sport University, 50933 Cologne, Germany; l.rappelt@dshs-koeln.de (L.R.); j.deutsch@dshs-koeln.de (J.-P.D.); l.donath@dshs-koeln.de (L.D.)

**Keywords:** IMU, velocity-based training, VBT, barbell speed, position transducer, sensor

## Abstract

The accurate assessment of the mean concentric barbell velocity (MCV) and its displacement are crucial aspects of resistance training. Therefore, the validity and reliability indicators of an easy-to-use inertial measurement unit (VmaxPro^®^) were examined. Nineteen trained males (23.1 ± 3.2 years, 1.78 ± 0.08 m, 75.8 ± 9.8 kg; Squat 1-Repetition maximum (1RM): 114.8 ± 24.5 kg) performed squats and hip thrusts (3–5 sets, 30 repetitions total, 75% 1RM) on two separate days. The MCV and displacement were simultaneously measured using VmaxPro^®^ and a linear position transducer (Speed4Lift^®^). Good to excellent intraclass correlation coefficients (0.91 < ICC < 0.96) with a small systematic bias (*p* < 0.001; η_p_^2^ < 0.50) for squats (0.01 ± 0.04 m·s^−1^) and hip thrusts (0.01 ± 0.05 m·s^−1^) and a low limit of agreement (LoA < 0.12 m·s^−1^) indicated an acceptable validity. The within- and between-day reliability of the MCV revealed good ICCs (0.55 < ICC < 0.91) and a low LoA (<0.16 m·s^−1^). Although the displacement revealed a systematic bias during squats (*p* < 0.001; η_p_^2^ < 0.10; 3.4 ± 3.4 cm), no bias was detectable during hip thrusts (*p* = 0.784; η_p_^2^ < 0.001; 0.3 ± 3.3 cm). The displacement showed moderate to good ICCs (0.43 to 0.95) but a high LoA (7.8 to 10.7 cm) for the validity and (within- and between-day) reliability of squats and hip thrusts. The VmaxPro^®^ is considered to be a valid and reliable tool for the MCV assessment.

## 1. Introduction

Velocity-based training (VBT) for strength and power conditioning has gained increasing interest in numerous sports [1]. Based on a strong relationship between the movement velocity and the relative load of the one repetition maximum (% 1RM), resistance training can be controlled by movement velocity [2,3]. This VBT approach enables a 1RM estimation based on the load–velocity relationships on a daily basis in real-time with an acceptable degree of accuracy compared with traditional 1RM testing (R^2^ = 0.954; standard error of the estimate (SEE) = 4.02%) [3,4]. These findings suggest that VBT enables a robust, non-invasive and highly sensitive method to estimate relevant strength training indicators such as the relative loading intensity (% 1RM), the maximum strength (1RM) [5] or the level of effort and neuromuscular fatigue during a training set [2]. In addition, VBT allows the application of resistance training monitoring without excessive exhaustion [2]. If the mean concentric (barbell) velocity (MCV) drops below a certain level (% of velocity loss), the set is stopped in favor of the next set, still having a few repetitions in reserve until failure [2].

In order to assess acute or training-induced changes of strength performance measures via VBT, an accurate and reliable measurement of the MCV is necessary [6]. As a minimal measurement error (reliability) is critically important to sports research [7], the validity and reliability of such sensors should be carefully examined [1]. Previous research underpins that linear position transducers provide high accuracy for an MCV assessment [8]. In conclusion, linear position transducers can be considered to be the gold standard in the field of assessing the MCV [8,9,10]. Recently, inertial measurement unit (IMU) sensors have also been used to measure the MCV [1,11]. Apart from this, IMUs are frequently used for: (i) gait analysis [12,13]; (ii) pedestrian navigation tracking [14,15,16,17,18,19,20,21]; (iii) ankle rehabilitation [22]; (iv) foot pose estimation [23]; (v) foot clearance estimation [24]; game play monitoring [25] and (vi) foot strike detection during running [26]. In general, IMU devices use a data fusion of a three-axis gyroscope, three-axis geomagnetic sensor and three-axis accelerometer to provide robust distortion-free and refined absolute position and orientation vectors [13]. These data fusion approaches are frequently conducted via Kalman filters [12,18,20], clustering algorithms [21] and/or hidden Markov models [17]. In the context of MCV measurements, IMU-based sensors have an advantage compared with linear position transducers [1]. Linear position transducers as cable-extension devices can be perceived as impractical within daily training routines and are prone to cable defects [1,11] thus the employment of IMU-based sensors can be regarded as the most feasible and easily applicable solution [1]. The IMU sensor is mounted to the barbell without the need for a fragile cable-extension or complex video-based solutions [11]. Accordingly, IMUs are particularly well-suited for practical use during daily training apart from intervention studies. The commercially available VmaxPro^®^ sensor is such an IMU-based device. The manufacturer promises a valid and reliable measurement of the MCV and barbell displacement [27]. So far, there are no independent scientific-based data on this device. As the VmaxPro^®^ only has to be placed on the barbell using the built-in magnet, the effort required in daily training is significantly lower compared with linear position transducers or video-based measurement systems. Therefore, the VmaxPro^®^ appears to be a practical and easy-to-use solution for the MCV and barbell displacement measurements in daily training.

Against this background, the objective of our study was to quantify the validity of a commercially available IMU sensor compared with a valid, reliable and accurate linear position transducer. Although within-day reliability is crucial for monitoring acute strain (velocity loss) during resistance training [2], between-day reliability plays an important role in detecting chronic performance developments [7]. Therefore, the absolute and relative within-day and between-day reliability indices of both devices were also assessed.

## 2. Materials and Methods

### 2.1. Participants

An a priori conducted power analysis (α = 0.05; two-tailed; study power (1-β-error) = 0.95; intraclass correlation coefficient = 0.75) performed using G*Power (Version 3.1.9.6, University of Kiel, Kiel, Germany) determined a required sample size of *n* = 16. Assuming a low to moderate dropout rate, 19 resistance training-experienced males (age: 23.1 ± 3.2 years, height: 1.78 ± 0.08 m, body mass: 75.8 ± 9.8 kg, body fat: 14.8 ± 5.6%, squat 1RM: 114.8 ± 24.5 kg, hip thrust 1RM: 171.1 ± 26.0 kg) were enrolled in this randomized controlled crossover validity and reliability trial. All participants had a minimum of two years of resistance training experience, were at least 18 years of age, did not present any health impairments and had not suffered from any neuromuscular or skeletal impairments in the past six months. Prior to the testing procedure, all participants were accustomed to the required equipment, protocol and exercises. In addition, the required technique was visually controlled by a certified and experienced strength and conditioning coach (during all lab visits). The participants were asked to refrain from any strenuous activity 24–48 h prior to each testing session and day. The study protocol complied with the Declaration of Helsinki, was approved by the local ethical committee (176/2020: Ethical Commission, German Sport University, Cologne) and fulfilled the international ethical standards [28,29]. All participants signed written informed consent after receiving all relevant study information.

### 2.2. Study Design

Each participant underwent 4 lab visits: (1) familiarization; (2) 1RM testing; (3 and 4) validity and reliability assessment. Lab visits 1 and 2 were 48–72 h apart. Lab visits 2, 3 and 4 were interspersed by one week. A standardized warm-up protocol was performed prior to each familiarization and testing day consisting of 5 min self-selected dynamic stretching and joint mobilization exercises followed by two warm-up sets of ten reps at 40% 1RM and five reps at 60% 1RM, respectively. During the familiarization session, multiple (3–4) sets of squats (SQ) and hip thrust (HT) exercises were performed with moderate loads (approximately 60% 1RM, based on the 1RM reported by the participants). In order to minimize fatigue effects, only two major exercises that addressed both the anterior (SQ) and posterior chain (HT) of the lower body muscles were selected. The procedure used for assessing the SQ and HT 1RM (lab visit 2) is described in detail by Kraemer and colleagues [30]. Briefly, a warm-up set of five repetitions at 50% of the predicted 1RM was performed followed by four repetitions at 80% of the presumed 1RM. Subsequently, a single repetition at 90% of the presumed 1RM was performed. Further (single repetition) sets were performed with slightly increased loads (0.5 to 5.0 kg). This procedure continued until the 1RM or failure was reached. In order to avoid fatigue, the 1RM was achieved (for all participants) at the latest during the third or fourth attempt. The applied 1RM test protocol has a high intraclass correlation coefficient (0.998) and a low technical error of measurement (1.66%) [30]. During the SQ, a depth of the hips below the top of the patella was required, which was visually controlled by a certified and experienced strength and conditioning coach. At the third and fourth lab visits, the participants completed a total of 30 repetitions per day (separated into 3–5 sets at 75% 1RM with a 3 min set break in between) of the SQ and HT exercises. The participants were encouraged to perform concentric actions explosively at a maximal intended concentric velocity. In order to control the potential circadian effects on the performance, all measurements were intraindividually conducted at similar times of the day for each participant.

### 2.3. Data Collection

**Figure 1:**
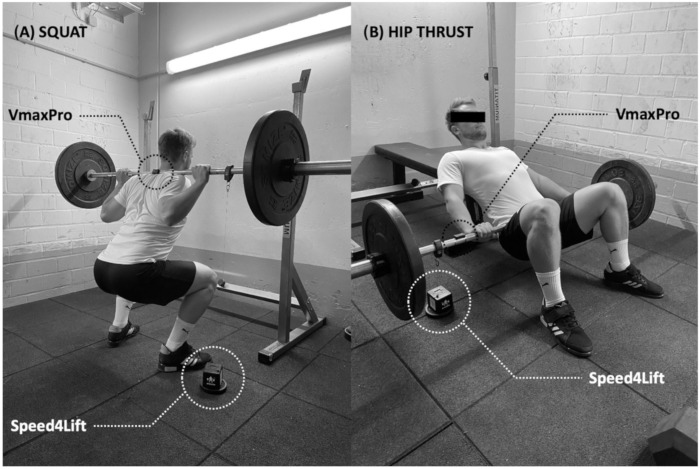
Exemplary execution of the squat (**A**) and hip thrust (**B**) measurement setup. The VmaxPro^®^ sensor is marked in black and the Speed4Lift^®^ sensor in white.

The MCV and barbell displacement were independently but simultaneously measured (Figure 1) with the Speed4Lift^®^ (Madrid, Spain; S4L) and the VmaxPro^®^ (Blaumann & Meyer, Sports Technology UG, Magdeburg, Germany; VMP) sensors. Therefore, both devices separately collected the MCV and barbell displacement during the same repetitions. The S4L is proven to be an accurate, valid and reliable (SE < 0.01 m·s^−1^; CV < 1.8%; ICC > 0.999) linear position transducer [31], which consists of a cable-extension linear position transducer attached to the barbell. Data were directly recorded by the differentiation of the displacement data with respect to time at a sampling rate of 1000 Hz. Visibility was enabled via a Wi-Fi connection with a smartphone (iPhone 10, Apple, Silicon Valley, CA, USA) using S4L application v.4.1. The cable of the S4L was vertically attached to the left side of the barbell using a Velcro strap.

The VMP is a commercially available wireless IMU that includes a three-axis accelerometer, gyroscope and magnetometer [27]. Data were directly recorded by the integration of the vertical acceleration with respect to time at a sampling rate of 1000 Hz through a Bluetooth (65 Hz) connection with a smartphone (iPhone 10, Apple, Silicon Valley, CA, USA) using the VMP application. According to the manufacturer, the VMP has been shown to be reliable and valid when measuring the peak velocity, MCV and barbell displacement during squats, bench presses, deadlifts and snatches [27]. Prior to each measurement, the VMP was calibrated according to the manufacturer’s specifications. For this purpose, the VMP was placed (in a specified order) on all six sides on a plane horizontal ground. Based on this calibration, the three-dimensional local coordinate system of the IMU (for the measurement of acceleration, angular rates and magnetic field vectors) could be aligned with the outer VMP sensor casing [32]. Post-calibration, the VMP sensor was placed on the barbell (using a built-in magnet) so that the local coordinate axes were parallel to the transverse, frontal and sagittal plane, respectively.

For further data processing, all reps at 75% 1RM were used for validity testing (S4L vs. VMP). To avoid fatigue effects, only the first two sets on each day (at 75% 1RM) were used for the within- (set 1 vs. set 2) and between-day (lab visits 3 vs. 4) reliability analyses. Thereby, the maximum MCV and barbell displacement of the first 3 reps were analyzed.

### 2.4. Statistics

All data are presented as group means ± standard deviation or with 95% confidence intervals, respectively. The collected data were examined for normal distribution and variance homogeneity. Several one factorial (device: S4L vs. VMP) repeated measure analysis of variance (rANOVA) were computed separately for each outcome measure (MCV and barbell displacement). In addition, several 2 (device: S4L vs. VMP) × 2 (within-day time: set 1 vs. set 2 or between-day time: lab visits 3 vs. 4) rANOVA were performed for the MCV and barbell displacement (for both devices). The rANOVA effect sizes were given as η_p_^2^ with values ≥ 0.01, ≥ 0.06 or ≥ 0.14 indicating small, moderate or large effects, respectively [33]. In case of significant rANOVA effects, Bonferroni post-hoc tests were subsequently computed. Standardized mean differences (SMD) as a measure of the pairwise effect size estimation were also calculated (SMD; trivial: SMD < |0.2 |; small: |0.2 |  ≤ SMD < |0.5 |; moderate: |0.5 |  ≤ SMD < |0.8 |; large SMD  ≥ |0.8 |) [33]. The agreement between both measurement devices (S4L vs. VMP) and (within- and between-day) reliability were analyzed by calculating the systematic bias (mean difference between the devices/sets/days) and the limit of agreement (LoA: 1.96 × standard deviation of the difference between both devices), considering a 95% random error component [7] and by plotting several Bland–Altman plots [34]. A typical error of measurement (TE), a standard error of measurement (SEM), a coefficient of variation (CV) and the intraclass correlation coefficients (ICC) were calculated [7]. ICCs were rated as excellent (0.9 to 1), good (0.74 to 0.9), moderate (0.4 to 0.73) and poor (0 to 0.39) [35]. For better comparability with other VBT-related validation studies, the Pearson correlation coefficient (CC) was also calculated [7]. The statistical analyses were conducted using R (version 4.0.5) and RStudio (version 1.4.1106) software.

## 3. Results

### 3.1. Validity

**Figure 2:**
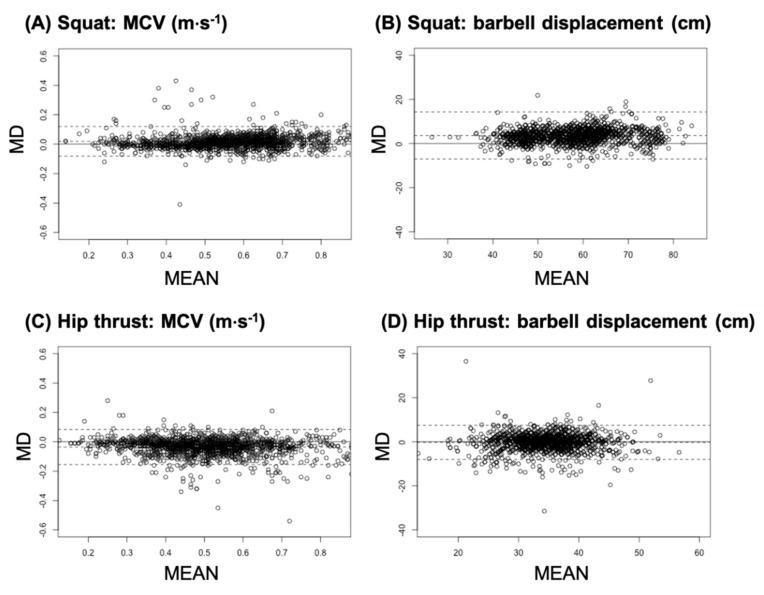
Bland–Altman plots (MD: mean difference between both devices; MEAN: average of both devices) for the validity of the Speed4Lift^®^ (S4L) vs. the VmaxPro^®^ (VMP) sensors for the mean concentric velocity (MCV; **A**,**C**) and barbell displacement (**B**,**D**). Squat (SQ; **A**,**B**) and hip thrust exercises (HT; **C**,**D**) are displayed separately.

The rANOVA revealed a significant effect of the MCV between the devices (S4L and VMP) for both the SQ (*p* < 0.001; η_p_^2^ = 0.50) and the HT (*p* < 0.001; η_p_^2^ = 0.26). Based on post-hoc tests, the SQ revealed a significant lower MCV (*p* < 0.001; MD = 0.01 ± 0.04 m·s^−1^; SMD = 0.11) for the VMP (0.52 ± 0.12 m·s^−1^) compared with the S4L (0.53 ± 0.12 m·s^−1^). In contrast, the HT revealed a significant higher MCV (*p* < 0.001; MD = −0.03 ± 0.05; SMD = −0.28) for the VMP (0.49 ± 0.11 m·s^−1^) compared with the S4L (0.45 ± 0.11 m·s^−1^). The rANOVA revealed a significant effect of the barbell displacement between the S4L and the VMP during the SQ (*p* < 0.001; η_p_^2^ = 0.10). Subsequently computed post-hoc tests revealed significant lower barbell displacement values (*p* < 0.001; MD = 3.37 ± 3.38 cm; SMD = 0.36) for the VMP (55.5 ± 9.2 cm) compared with the S4L (58.8 ± 9.5 cm). In contrast, the rANOVA revealed no significant effect (*p* < 0.78; η_p_^2^ < 0.001; MD = 0.03 ± 3.34 cm; SMD = 0.01) for the barbell displacement during the HT between the VMP (32.8 ± 4.7 cm) and the S4L (32.9 ± 5.0 cm). The ICC and CC revealed a good to excellent validity between both devices for the MCV and barbell displacement during both the SQ and HT (Table 1). In addition, the TE, CV and SEM of the MCV and barbell displacement were low during the SQ and HT (Table 1). For a descriptive validity agreement analysis between the S4L and VMP, Bland–Altman plots are depicted in Figure 2. Although the MCV measurements were characterized by a low level of agreement, the barbell displacement limits of agreement were considerable higher (Table 1).

### 3.2. Between- and Within-Day Reliability

The rANOVA revealed a significant effect of both the MCV (*p* = 0.029; η_p_^2^ = 0.14) and the barbell displacement (*p* = 0.044; η_p_^2^ = 0.13) for S4L during the between-day assessment of the HT. Based on the post-hoc tests, the second day revealed significantly higher MCV (*p* = 0.029; MD = −0.04 ± 0.10 m·s^−1^; SMD = −0.38) and barbell displacement (*p* = 0.044; MD = −0.02 ± 0.06 cm; SMD = −0.37) values during the between-day comparisons. Apart from this, no significant effects (*p* > 0.100; η_p_^2^ < 0.14) were found for any parameter in both devices during within- and between-day reliability testing with only a trivial to small SMD (< 0.17). Based on the ICC and CC, both devices showed good to excellent (within- and between-day) reliability for all SQ parameters (see Table 2). In addition, the HT revealed moderate to excellent (within- and between-day) reliability for all HT parameters (see Table 2). Although the MCV (within- and between-day) reliability measurements were characterized by a low level of agreement, the barbell displacement limits of agreement were considerable higher (see Table 2).

## 4. Discussion

This is the first study that assessed the validity and absolute and relative within- and between-day reliability of the commercially available IMU-based VmaxPro^®^ (VMP) sensor in comparison with a highly valid [31] linear position transducer (Speed4Lift^®^; S4L). Based on a good to excellent intraclass correlation (ICC) and a low standard error of measurement (SEM), we observed good to excellent validity for both the mean concentric velocity (MCV) and the barbell displacement during squat and hip thrust exercises between the VmaxPro^®^ and the Speed4Lift^®^ sensors. Both devices (Speed4Lift^®^ and VmaxPro^®^) additionally showed a good to excellent within- and between-day reliability (based on the ICC and CC) for the squat exercise with respect to both the mean concentric barbell velocity and the barbell displacement. The mean concentric velocity and the barbell displacement of the hip thrust exercise revealed (based on the ICC) a good within-day reliability and a moderate between-day reliability for both devices (Speed4Lift^®^ and VmaxPro^®^). The mean concentric velocity during squats and hip thrusts revealed low limits of agreement [7,34], which indicates a high validity and (within- and between-day) reliability. In contrast, the barbell displacement revealed high limits of agreement for both squats and hip thrusts, which indicates insufficient accuracy in terms of validity and (within- and between-day) reliability.

A recently conducted systematic review [1] summarized the evidence on the validity and reliability data of eight commercially available IMU devices for measuring barbell velocity: The Beast^®^ (Beast Technologies, Brescia, Italy) [9,10,36], the Gyko sport^®^ (Microgate, Bolzano, Italy) [37] and the PASCO^®^ (Roseville, CA, USA) [38] sensors can be considered to be valid (SEM = 0.01 to 0.18 m·s^−1^; CC = 0.79 to 0.93). Compared with these previously validated IMU-based sensors [1], the validity of the current examined VmaxPro^®^ sensor appears to be comparable or even superior (SEM: 0.01 to 0.18 vs. 0.01 to 0.02 m·s^−1^; CC: 0.79 to 0.93 vs. 0.91 to 0.96). Based on the calculated limits of agreements [7,34] (0.10 to 0.12 m·s^−1^; see Table 1), the estimation of load (% 1RM) from the velocity measures would imply a discrepancy of <5% 1RM [4,5] between the VmaxPro^®^ and the Speed4Lift^®^ sensor. As the 1RM is characterized by a day-to-day variance of up to 18% [6,39], a discrepancy < 5% appears to be acceptable for daily training practices.

The within-day reliability of the previously examined IMU sensors, such as the Beast^®^ (Beast Technologies, Brescia, Italy) [9,10,36], the Gyko sport^®^ (Microgate, Bolzano, Italy) [37] and the PASCO^®^ (Roseville, CA, USA) [38], were classified as adequate (ICC = 0.36 to 0.99; CV up to 35%) [1]. Thus, the (within-day) reliability of these previously examined IMU-based sensors [1] vs. the VmaxPro^®^ sensor (ICC: 0.36 to 0.99 vs. 0.56 to 0.95; CV: < 35.0% vs. < 7.5%) trends in favor of the VmaxPro^®^ sensor. In line with these findings, the within- (and also the between-) day reliability of the used linear position transducer (Speed4Lift^®^) did not notably differ from the examined VmaxPro^®^ sensor. In addition, the calculated limits of agreement for the within- and between-day reliability (see Table 2) resulted in an error < 5% during the velocity-based 1RM estimation in line with the validity data. Accordingly, the MCV measurement of the VmaxPro^®^ can be considered to be reliable.

Although the barbell displacement is especially interesting for strength and conditioning coaches [40], it has not been assessed in previous IMU-related research. Based on the intraclass correlations, we observed a good validity for the barbell displacement during squats and hip thrusts (see Table 1) of the VmaxPro^®^ sensor compared with the used linear position transducer (Speed4Lift^®^). Considering the limits of agreements, this validity based on the intraclass correlations has to be challenged [7,34]. The limits of agreements (see Table 1) of up to 10.7 cm corresponded with up to 25% of the total barbell displacement during squats and hip thrusts. As the achievable load (in % 1RM) is directly determined by the squat depth (corresponding with the barbell displacement) [40], these limits of agreements appear to be too high for monitoring the barbell displacement during the daily training process.

The within- and between-day reliability assessments revealed moderate to excellent intraclass correlations (of the VmaxPro^®^ sensor) for the barbell displacement during squats and hip thrusts. In addition, the within- and between-day reliability (based on the ICC) of the used linear position transducer (Speed4Lift^®^) did not notably differ compared with the examined VmaxPro^®^ sensor (see Table 2). Similar to the validity evaluation, the limits of agreements for the within- and between-day reliability assessments (up to 10.8 cm; see Table 2) appear to be also too high for monitoring the barbell displacement during the daily training process. Accordingly, the IMU-based VmaxPro^®^ sensor did not appear to be suitable for measuring the barbell displacement on a valid and reliable basis.

As the VmaxPro^®^ is a commercially available product, the manufacturer provides only restricted information on the technical specification, configuration, settings, data filtering, algorithms and integration (of acceleration values) [27]. Furthermore, raw data cannot be exported either. However, this information is not relevant for trainers, coaches and athletes. In the daily training process and during performance testing, only the resulting MCV provided by the VmaxPro^®^ device is required. Therefore, the following practical applications apply. A valid measurement of the mean concentric barbell velocity builds the foundation of velocity-based strength training [1,2,3]. The mean concentric velocity enables a robust, non-invasive and highly sensitive method to estimate the relative loading intensity (% 1RM) or maximum strength (1RM) [5]. In order to detect training-induced performance adaptations via these velocity-based 1RM estimations [2,5], a promising between-day reliability is required [7]. For the velocity-based estimation, the level of effort and neuromuscular fatigue incurred during a training set [2] and proper within-day reliability is additionally required [7]. As the findings of the current results revealed the validity and (within- and between-day) reliability of the commercially available IMU-based VmaxPro^®^ sensor for measuring the mean concentric barbell velocity, the VmaxPro^®^ can be used for such velocity-based approaches. The VmaxPro^®^ sensor is mounted to the barbell without the need for a cable-extension or complex operations such as video-based solutions [11]. Therefore, practitioners looking for the most feasible solution to monitor movement velocity during resistance training should consider the VmaxPro^®^ sensor as a valid and reliable alternative to traditional linear position transducers.

## 5. Conclusions

In summary, (i) linear position transducers such as the Speed4Lift^®^ are suggested as a gold standard in the field of assessing the mean concentric velocity and barbell displacement [8,9,10]; (ii) the IMU-based VmaxPro^®^ sensor showed good to excellent validity with relatively low limits of agreements for the mean concentric velocity during squats and hip thrusts; (iii) there was a comparable (within- and between-day) reliability with previously validated linear position transducers (Speed4Lift^®^) for the mean concentric velocity during squats and hip thrusts; (iv) there were low limits of agreements for the mean concentric velocity during (within- and between-day) reliability testing for squats and hip thrusts and (v) in contrast, the barbell displacement validity and (within- and between-day) reliability measurements of squats and hip thrusts were characterized by too high limits of agreement despite good intraclass correlations. In conclusion, the commercially available IMU-based VmaxPro^®^ sensor can be considered to be a valid and reliable measurement tool for the mean concentric velocity. For the barbell displacement measurements, the high limits of agreement seriously restrict the practical use of the IMU-based VmaxPro^®^ sensor.

## Figures and Tables

**Table 1:** Validity of the mean concentric velocity (MCV) and barbell displacement between the Speed4Lift^®^ (S4L) and VmaxPro^®^ (VMP) sensors for squat (SQ) und hip thrust (HT) exercises.

Exercise	Parameter	TE (%)	CV (%)	ICC (95% CI)	CC (95% CI)	LoA	SEM
SQ	MCV (m·s^−1^)	5.6	7.6	0.94 0.92–0.95)	0.96 *** (0.95–0.96)	0.1	0.01
	Barbell displacement (cm)	5.9	5.9	0.88 (0.43–0.95)	0.84 *** (0.83–0.85)	10.69	1.18
HT	MCV (m·s^−1^)	9.2	11.2	0.85 (0.70–0.92)	0.91 *** (0.91–0.92)	0.12	0.02
	Barbell displacement (cm)	7.2	10.2	0.76 (0.73–0.78)	0.76 *** (0.74–0.78)	7.78	1.64

Typical error of measurement (TE), coefficient of variation (CV), intraclass correlation coefficient (ICC), Pearson correlation coefficient (CC), limit of agreement (LoA) and standard error of measurement (SEM). Pearson correlation significances are given as *** *p* < 0.001.

**Table 2:** Within- and between-day reliability of the mean concentric velocity (MCV) and barbell displacement for Speed4Lift^®^ (S4L) and VmaxPro^®^ (VMP) sensors for squat (SQ) und hip thrust (HT) exercises.

Device	Exercise	Reliability	Parameter	TE (%)	CV (%)	ICC (95% CI)	CC (95% CI)	LoA	SEM
S4L	SQ	Within-day	MCV (m·s^−1^)	3.3	4.4	0.91 (0.78–0.97)	0.92 *** (0.80–0.97)	0.04	0.01
			Barbell displacement (cm)	3.8	5.5	0.94 (0.85–0.98)	0.94 *** (0.84–0.98)	6.58	0.82
		Between-day	MCV (m·s^−1^)	8.6	12.3	0.75 (0.56–0.87)	0.75 *** (0.85–0.87)	0.21	0.05
			Barbell displacement (cm)	6.7	9.3	0.78 (0.61–0.88)	0.79 *** (0.61–0.89)	11.67	2.8
	HT	Within-day	MCV (m·s^−1^)	3.3	4.5	0.93 (0.83–0.97)	0.93 *** (0.83–0.97)	0.05	0.01
			Barbell displacement (cm)	4.8	6.9	0.71 (0.39–0.88)	0.71 *** (0.37–0.88)	5.07	1.40
		Between-day	MCV (m·s^−1^)	12	15.9	0.56 (0.22–0.74)	0.56 *** (0.26–0.76)	0.2	0.72
			Barbell displacement (cm)	10.3	13.9	0.49 (0.19–0.71)	0.52 ** (0.208–0.735)	10.68	3.87
VMP	SQ	Within-day	MCV (m·s^−1^)	3	4.4	0.88 (0.71–0.95)	0.87 *** (0.69–0.95)	0.05	0.09
			Barbell displacement (cm)	4.5	6.5	0.91 (0.79–0.97)	0.91 *** (0.78–0.96)	7.29	1.11
		Between-day	MCV (m·s^−1^)	6.9	9.9	0.82 (0.67–0.90)	0.81 *** 0.66–0.90)	0.16	0.04
			Barbell displacement (cm)	5.6	8	0.83 (0.69–0.91)	0.83 *** (0.69–0.91)	9.43	1.98
	HT	Within-day	MCV (m·s^−1^)	5.3	7.4	0.80 (0.56–0.92)	0.81 *** (0.57–0.93)	0.09	0.02
			Barbell displacement (cm)	6	8.7	0.58 (0.18–0.82)	0.57 ** (0.16–0.82)	6.4	2.19
		Between-day	MCV (m·s^−1^)	10.4	14.9	0.55 (0.25–0.75)	0.55 ** (0.24–0.75)	0.21	0.07
			Barbell displacement (cm)	9.3	13.3	0.41 (0.07–0.66)	0.41 * (0.07–0.66)	10.75	4.23

Typical error of measurement (TE), coefficient of variation (CV), intraclass correlation coefficient (ICC), Pearson correlation coefficient (CC), limit of agreement (LoA) and standard error of measurement (SEM). Pearson correlation significances are given as *** *p* < 0.001; ** *p* < 0.01 and * *p* < 0.05.

## Data Availability

All data are presented in the manuscript. Additionally, data are available on request from the corresponding author.
